# The University of Maryland Dental Department

**Published:** 1889-01

**Authors:** W. N. Murphy

**Affiliations:** La Grange, Texas.


### Editorial, Etc.

The University of Maryland Dental Department.—Having noticed for several months the correspondence between Prof. F. J. S. Gorgas, Dean of the Dental Department of the University of Maryland, and Drs. Waters, Levy and others, I desire to add what little information I have which I hope will be of some interest to the readers of the Journal. During the session of 1885 and 1886, I was a student at the Maryland University, Dental Department, and some time during the session, either just before or just after the holidays, I called at the office of Prof. Gorgas, the dean, in company with Mr. Robertson, of Canada, and Mr. Bemis, of Boston, all of us asking to be admitted to the graduating class on five years' practice and one full session which we were then attending, whereupon we were refused. The writer appreciated the firmness of the dean in carrying out to the letter the resolutions of the National Association of Dental Examiners, and the great interest he feels in the progress of dental education. At the next regular session, we all returned and graduated.

To the honor and credit of Prof. Gorgas as well as the other members of the faculty, I can truthfully say that no man labors harder for the interests of the dental profession than Prof. Gorgas, who is now the oldest man at the helm of dental education with one exception, Prof. Taft. I do not know Drs. Waters and Levy, nor do I know that their names have been sounded very far from their respective homes, nor do I know that my own name has ever gone beyond the limits of Fayette county, though I do know that the name of Prof. Gorgas has been dropped from the lips of almost every tongue to ring through the ears of nearly every nationality. I do hope that Drs. Waters and Levy had no intention of injuring Dr. Gorgas or the Dental Department of the Maryland University, though they have never said so, but if they did, the honors that Prof. Gorgas has so justly and faithfully born well up into the evening of life are more than two or three men can tear down.

W. N. Murphy, D. D. S.,
La Grange, Texas.

